# An Ethics Perspective on Transcranial Magnetic Stimulation (TMS) and Human Neuromodulation

**DOI:** 10.1155/2006/791072

**Published:** 2006-11-27

**Authors:** Judy Illes, Marisa Gallo, Matthew P. Kirschen

**Affiliations:** ^1^Stanford Center for Biomedical EthicsStanford UniversityCAUSA; ^2^Department of RadiologyStanford UniversityCAUSA; ^3^Program in NeuroscienceStanford UniversityCAUSA

## Abstract

This paper concerns the ethics of human neuromodulation using transcranial magnetic stimulation (TMS). We examine the challenges of modulating the brain with TMS through the research ethics lens and in clinical medicine for treating frank pathology, primarily in psychiatric diseases. We also consider contemporary issues raised in the neuroethics literature about managing unexpected findings, and relate these to TMS and to other frontier neurotechnology that is becoming openly available in the public domain. We argue that safety and informed consent are of paramount importance for TMS, but that personal values and sociocultural factors must also be considered when examining the promise of this technology and applications that ought to be highlighted for extra precautions.

## 1. The dynamic evolution of TMS research

Transcranial magnetic stimulation (TMS) involves a powerful and rapidly changing electrical current transmitted through a coil that is placed on the scalp. It produces a magnetic field that passes unimpeded through the skull and induces a weaker electrical current in the brain [109] that transiently disrupts neural circuits at the stimulation site. The growing body of literature on TMS (Fig. 1) suggests that TMS offers several advantages over other non-invasive neuroimaging techniques in the study of normal neural functioning. Techniques like functional magnetic resonance imaging (fMRI), positron emission tomography (PET), magnetoencephalography (MEG) and electroencephalography (EEG) rely on correlations to establish brain-behavior relationships. Functional MRI, for example, correlates changes in hemodynamic signal with cognitive behavior. TMS, on the other hand, characterizes behavioral changes with selective disruption of normal neural signaling, revealing neural structures necessary for normal behavioral and cognitive functions. Compared to patient lesion studies – that is, studying patients with strokes or tumors – TMS has some advantages since compensatory mechanisms and functional rewiring over time can obscure understanding of the discrete function of the originally damaged tissue. In examining fundamental issues of research ethics covered in The Belmont Report issued by the National Commission for the Protection Human Subjects in Biomedical and Behavioral Research (1979), unique issues of safety, informed consent and disclosure stand out for TMS.

### 1.1. Considerations of safety

Single-pulse TMS is thought to be extremely safe and has proven to be a valuable tool for investigating normal human neurophysiology. It has been used effectively over the past 20 years to help us gain detailed knowl-

ISSN 0953-4180/06/$17.00 © 2006 – IOS Press and the authors. All rights reserved

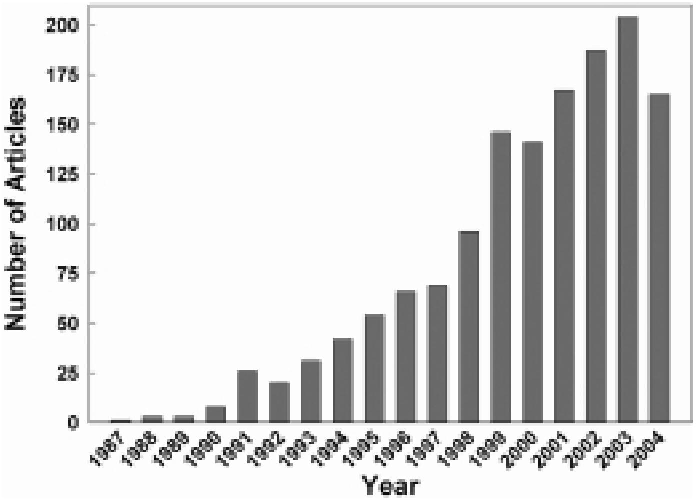

Fig. 1. Number of TMS articles published in the scientific literature per year between January 1985 and July 2004.

edge about brain-behavior relationships in such areas as motor systems [16,19,96,111], visual and perceptual processing [7,50,51,68,93], language [30,33,92], verbal working memory [28,53,82] and memory guided saccades [15,26,35,42,59,83,85,86,91]. Several thousand individuals have participated as normal controls in these experiments with very few adverse reactions. A small percentage of research subjects describe non-specific symptoms like headache, nausea [99] or tinnitus after several hundred TMS pulses during a single experimental session, but no serious adverse reactions have been reported.

Enough single-pulse TMS safety studies have been conducted on both animals and humans to confidently state that there are no known short or long-term sequelae to TMS stimulation [17,21,65,67]. In one study, non-human primates receiving 7,000 maximum intensity single TMS pulses delivered in daily increments over thirty days demonstrated no short or long term deficits of higher cerebral functions [112]. A subsequent study administered 1,200 to 3,800 stimuli at 5–20 Hz over the visual cortex of eleven healthy volunteers and did not provide any evidence of pathological changes on contrast MRI or diffusion scans, demonstrating that TMS does not adversely affect the blood-brain barrier or induce localized edema [90]. In lobectomy specimens obtained from two epileptic patients following repetitive TMS, no structural brain damage was found [61].

Compared to single-pulse TMS, repetitive TMS (rTMS) is a more powerful tool, capable of making a pronounced and possibly irreversible impact on neural functioning. It has been reported to induce seizures in a small percentage of healthy subjects [20,43,109], and is therefore more likely to have longer-term effects than single pulse TMS on neural functioning. In an effort to establish safe parameters for rTMS, Pascual-Leone et al. [94] evaluated the effect of varying frequency and intensity of rTMS on cortical excitability in healthy volunteers. Adverse reactions such as headaches, visual disturbances, vertigo, weakness, and paresthesias were not experienced by subjects. Blood pressure, pulse, and ECG levels remained unchanged after stimulation. One subject experienced a seizure after three stimuli to the left motor cortex. It was later discovered that the subject had elevated prolactin levels and a family history of seizures. Other studies have also found a rise in hormones, specifically thyroid stimulating hormone (TSH), following TMS [39,103]. A second subject in Pascual-Leone et al.'s study experienced tinnitus in the left ear following rTMS, which lasted less than 30 minutes [94]. Seizures induced by TMS are estimated to occur in about 1 out of 1000 TMS and rTMS subjects [3,109].

Studies of TMS on children have identified side effects such as scalp discomfort, hand weakness, headache, neck and arm pain, and arm tingling [34,40, 80]. After surveying 28 TMS studies involving over 850 children, Gilbert et al. [40] recommended that institutional review boards classify TMS as a minimal risk procedure, despite the noted adverse effects.

In 1996, an international workshop was held to delineate the risks of rTMS and establish relevant guidelines [109]. Workshop topics covered stimulation parameters, physiological monitoring, neuropsychological monitoring, training and qualifications of rTMS operators, medical management of seizures, management of psychological consequences of seizures, and contraindications of rTMS. With a decade of experimentation in humans since Barker et al.'s [5] first description of the TMS device in 1985, and increasing uses of rTMS, the timeliness of this meeting was indisputable. Yet, for any frontier neurotechnology like rTMS whose discovery and further evolution may have a rapid far-reaching impact in both research and clinical medicine, it is essential that such guidelines undergo continuous review and be as dynamic as the technology itself.

### 1.2. *Informed consent and disclosure*

In all biomedical research, potential benefits must outweigh the risks. As an evolving technology for the study of neural functioning, the scientific community is constantly learning more about the effects of TMS, both short and long term, on the human brain. However, given the state-of-the-art of the technology, an important question is whether enough is known about the way TMS interferes with normal brain functioning to truly obtain an informed consent from research volunteers. As we discussed above, single-pulse TMS is considered to be relatively safe. Nonetheless, many questions remain as to the nature of basic physiology of TMS-induced effects on the brain. It is evident from almost all studies that behavior on specific tasks returns to baseline post-stimulation, but it is possible that current tasks are not sensitive enough to uncover deficits that might remain, or that functions that might remain impaired (e.g., attention or speed of information processing) are not tested.

The remote effects of TMS throughout the whole brain are also still being explored. It is not yet known, for example, whether the effect of a single TMS pulse is confined to a small intended region of cortex, or if other cortical and sub-cortical structures are affected through either stray magnetic waves or magnetic waves that propagate sub-clinically through the vast neuronal interconnections of the human brain [8]. Interleaved TMS-fMRI studies [9,12,13,88], although not without technical complications [98,102], are currently being used to further our understanding of both direct and indirect effects of TMS, including the physical properties of TMS inside the human brain by mapping TMS fields in an MR scanner [10,11] and measuring current densities using depth EEG electrodes [108].

Since it is not possible to know all of the potential short- and long-term effects associated with either single-pulse or repetitive TMS, the informed consent process must provide full disclosure of all known significant risks and acknowledge the possibility of yet-unknown longitudinal effects. Such practice has precedent in the therapeutic domain, for example, where certain drugs may be prescribed to alleviate symptoms or manage diseases even though the molecular mechanisms are poorly understood. The mechanism of action of a high percentage of drugs approved by the FDA for use in the United States is listed as "unknown" in the Physicians Desk Reference (PDR), yet each of those drugs had to undergo several phases of rigorous clinical testing to ensure their safety and efficacy before obtaining FDA approval. The mechanism of how and where TMS affects the brain is becoming better understood all the time but, like pharmaceuticals, its exact mechanism of action still remains elusive.

## 2. Ethical challenges for TMS in the clinical domain

While it may be safe to stimulate healthy brain tissue, we have less information about the effects of TMS on abnormal brain tissue. This raises two major issues that we discuss here: the potential physical risks of stimulating already compromised brain tissue with TMS, and ethical challenges to using TMS clinically.

### 2.1. *Potential risks to functional connectivity of compromised tissue*

Some brain regions are particularly fragile and easily insulted while others are more resistant to stress and manipulation. Structures like the sensory-motor cortex have shown functional recovery after short periods of anoxia or seizure activity, whereas other structures like the hippocampus are more sensitive to stress and do not recover as quickly and might sustain more permanent damage [25,46].

Comprehensive studies have not yet been conducted looking at the effect of TMS on anatomically abnormal neural tissue, but single-pulse TMS has produced seizures in patients with predisposing brain lesions such as strokes, ALS and epilepsy [43]. The prevalence of incidental findings on research MRI scans raises the possibility of stimulating a region containing a tumor or vascular malformation unknowingly [57]. Although normal levels of neural activation have not yet been established, it is important to consider the ramifications of stimulating functionally compromised tissue. Thresholds for stimulation might vary in these tissues and we cannot dismiss the possibility that application of TMS might produce irreversible changes in connectivity and functionality.

## 2.2. Clinical challenges

The challenges in understanding why patients may elect to undergo an innovative procedure that is still unapproved by regulatory agencies in many countries, including the Food and Drug Administration in the United States, are not particularly exceptional. We can turn to examples from the surgery and experimental therapeutics literature, and even the alternative medicine literature, to understand patient motivation, especially when no better alternative seems to exist [18,24,62]. Such motivations can be driven by a number of factors: the sense that doctors 'know best', that refusing to take part would upset the doctor, that there is little risk associated with participation, or that people have a moral duty to be involved in such trials [31]. In a survey given to caregivers of patients with severe Alzheimer's dementia, for example, respondents believed that entering into a clinical trial provided some chance of improving or at least maintaining the patients' condition. Caregivers who declined participation noted the potential side effects of the drugs as the primary reason [29].

Aside from such relatively established ethical challenges to any to form of innovative therapy, exceptional challenges also exist. They lie, for example, in the timing and conventions of technology transfer. As Lisanby et al. [69] have articulated, "*The conventions in clinical trials on pharmaceutical agents in the treatment of psychiatric disorders do not translate perfectly to the study of TMS as a therapeutic intervention*."

In TMS trial designs, unique challenges are posed by issues of standardization, clinically appropriate targets for stimulation, navigation to targets, localization and effective controls and placebos. Further, while sham TMS [69] may be standard practice for experimental control, some have questioned whether the brain is completely unaffected by the contact of the magnetic field with the scalp and accompanying clicking noise [70]. Similar debate has surfaced about whether patients are able to discern between sham and TMS treatment [14] and fully comprehend the circumstances surrounding the procedures – an issue that brings us back to earlier discussions about informed consent. Finally, the pure ethical and experimental dilemma associated with targeting a region for TMS for a psychiatric illness to which a brain location has yet to be traced is inescapable. This latter issue is especially acute given significant variability in results of clinical trials of patients with depression, a major clinical focus for TMS [36,48,63,64,69,72,77,81,87,106,110], as well as mania [60,87], obsessive compulsive disorders [44, 49,73,76,97], schizophrenia [47,49,52,76,100], post traumatic stress disorder [22,45,74], Tourette Syndrome [38,84,113], Parkinson's Disease [14,71,101], epilepsy [27,78,104,105], ataxia [107], Pelizaeus-Merzbacher disease [89], blindness [23,95], optic atrophy [79], essential tremor [41] and migraine [2,4]. While variability in pre-existing pharmacology, gender, as well as other confounding factors such as age [32, 69] all complicate the interpretation of results, we note that compared to electroconvulsive therapy, TMS has less morbidity and appears to achieve greater precision in reaching deep brain structures [37,75].

## 2.3. Clinical ethics and TMS

From the clinical ethics literature, we can borrow broad principles such as *beneficence, non-maleficence, autonomy* and *justice* [6] for evaluating TMS trials and, in the future, for approved indications. Possible harm can be mitigated by disclosure of benefit and risk, coupled with autonomous decision-making (to the extent possible in vulnerable populations). Justice, the principle that refers to access to care for all people is relevant here, naturally, but extends well beyond the TMS application alone.

We can further enrich our analysis of the suitability of therapeutic approach for the individual patient by engaging a more patient-based framework using principles of casuistry [58]. This framework is based on concrete questions that guide ethical decision-making. For example, *medical indications* questions focus on the nature of a patient's medical condition (diagnosis, prognosis, acuteness, reversibility), and the available treatment options (goals, probabilities of success, benefits and harms, alternatives). In the case of TMS, the extent to which a patient's condition is debilitating and is refractory to other forms of treatment weighs heavily here. *Patient preference* questions personalize the assessment by considering the patient's desires and goals. The preference question also takes into consideration a competency for decision-making (informed consent, mental and legal competence), a key variable as we discussed above, for patients from vulnerable populations for whom TMS may be an option. *Quality of life* questions consider the traditional risk/benefit assessment of the treatment in terms of the patient's likelihood to return to normal life, deficits that could occur with treatment, and whether the patient would consider life undesirable without treatment. *Contextual features* questions draw attention to possible sources of bias in treatment decision-making such as familial beliefs, provider beliefs and cultural beliefs. They take into consideration the entire context of a patient who is referred to or is contemplating TMS intervention, including personal and sociocultural views.

Even with the level of detail for assessing decision-making that the casuistry model provides, there is still no way – nor would it be appropriate – to compute a score of the benefits versus risks to a single patient undergoing medical treatment, especially one such as TMS for which clinical uncertainty still abounds. Yet in describing this model for clinical ethics, Jonsen et al. [55] highlight the need for such a strong methodological approach. As they have stated, and as the new neuroethics literature asserts [54,56], when ethics and practice go hand in hand truly well reasoned analyses of the ethical dilemmas can take place.

## 3. Managing unexpected effects

It is theoretically possible to get unpredictable and unintentional behavioral responses when stimulating with TMS in either of the domains we have discussed above – research and clinical applications – or when potentially used in the context of personal choice for enhancing memory, attention or cognitive performance [66]. What are the ethical concerns if such an occurrence were to happen? If TMS research continues to evolve in the same manner as functional MRI studies [55], researchers will soon be routinely stimulating regions associated with higher order cognitive functions like emotion, humor and moral judgment. However unlikely, what would happen if a person shouted an obscenity, experienced hallucinations, flashbacks or vivid dreams, or made a confession to a criminal offense during or after a TMS session? These behaviors might be associated with the targeted region or be a result of collateral stimulation from a separate, unassociated region. We did not think that unexpected findings such as clinically significant structural abnormalities would be a problem on research brain MRIs, until we looked [57]. Although unusual manifestations of TMS have yet to be reported, the increasing prevalence of rTMS and precedent set by the trends in research make their discovery a true possibility.

## 4. Critical ethical thinking: From past to future neurotechnology

Using TMS as a non-invasive brain stimulation technique to study normal brain functioning by magnetically interfering with neural circuits qualifies as a slippery slope. Single-pulse TMS transiently disrupts neural networks for approximately 100 milliseconds with minimal risks and very few reported complications. Repetitive TMS has a more profound effect on the brain and affects cerebral functioning for a longer period of time. How big of a leap is it from rTMS to Cyberknife [1], a non-invasive stereotactic radiosurgery system, which affects function permanently by ablation of neural tissue? Knowing that the brain will functionally reorganize itself, would it be considered ethical to study normal brain function by ablating tissue with radiosurgery? The direct effects of such studies could easily be more controlled and therefore potentially more enlightening than current patient lesion studies and TMS experiments.

Both rTMS and Cyberknife are considered non-invasive procedures and both can theoretically have long-lasting effects on neural tissue and cognitive performance. Where do we draw the line as to how much we are willing to alter healthy brain tissue before deeming it unethical? We can mechanically induce strokes, pharmacologically initiate seizures, and genetically engineer tumors to grow in a variety of lab animals, but rats, mice and even non-human primates will never demonstrate the complex response to neurological disease as humans. Are we moving towards an era of performing lesion experiments on humans that formerly resided only in the province of animal research?

While TMS may be used beneficially to map functional brain regions before tumor surgery or to help victims obliterate memories for traumatic events like violent crime, it is also worth considering the potential commercial uses of this technology. TMS applications can impair memory in a confined experimental environment, but at high enough frequency, power and duration, rTMS could more permanently disrupt or suppress memory formation, decrease sexual drive or possibly repress the desire to lie. TMS or other similar technologies have already been portrayed in film for these purposes, as in the movie *Eternal Sunshine of the Spotless Mind* (Focus Features, 2004) in which the protagonist seeks to have his memories of past romance erased from his mind. While advertising and sales of memory erasure technology are still absent from the open marketplace, we must consider means of ensuring that all frontier neurotechnology is reserved for responsible research and clinical use, and questionable uses kept at bay. The technology must never be used in coercive ways. We must also consider policy in the context of how our individual values come into play. For example, should society have unfettered access to this technology if it becomes available in the open market? What will protect consumers – especially the openly ill or covertly suffering – from marketing lures that, in the hands of non-expert TMS entrepreneurs, may be no more effective than snake oil? How should science and society evaluate rTMS as a potential replacement for pharmacological therapy that might be mandated by the judicial system for treating severe pathological antisocial behavior as an alternative to incarceration?

## 5. Conclusion

This is a time of great promise. This is also a time of great challenge, as the capabilities of frontier neurotechnology have pushed the envelope of studying and modulating brain function to new depths and new breadth. As we move ahead in our explorations with TMS, we can rely on a strong history in research and clinical ethics to guide us. However, new challenges unique to TMS will require new consideration in basic research and clinical medicine – consideration of the new lines that, collectively as members of the scientific disciplines and individually as citizens of science, we will have to decide whether or not to cross.

## Acknowledgements

Supported by The Greenwall Foundation and NIH RO1 NS#045831. Thanks to Dr. Eric Racine and Dr. Brent Solvason for their valuable input.
